# A unusual case of multifocal pyogenic abscess formation following ERCP procedure

**DOI:** 10.1186/s12893-020-00759-y

**Published:** 2020-05-06

**Authors:** Fahed Merei, Galina Shapiro, Ibrahim Abu Shakra, Amitai Bickel, Samer Ganam, Maxim Bez, Eli Kakiashvili

**Affiliations:** 1 Department of Surgery A, Galilee Medical Center, Nahariya, Israel; 2 Medical Corps, Israel Defense Forces, Ramat Gan, Israel; 3 Faculty of Medicine in the Galilee, Bar-Ilan University, Safad, Israel

**Keywords:** Post-endoscopic retrograde cholangiopancreatography, Complications, Ascending cholangitis, Epidural abscess, Psoas abscesses

## Abstract

**Background:**

Endoscopic retrograde cholangiopancreatography (ERCP) is essential for managing biliary and pancreatic disorders. Infection is the most morbid complication of ERCP and among the most common causes of ERCP-related death.

**Case presentation:**

A 69-year-old man presented with right upper quadrant abdominal pain, obstructive jaundice and abnormal liver function tests. Ultrasound revealed cholelithiasis without bile duct dilation. After receiving intravenous antibiotics for acute cholecystitis, the patient was discharged. Two weeks later, an endoscopic ultrasound demonstrated gallstones and CBD dilation of up to 6.4 mm with 2 filling defects. An ERCP was performed with a papillotomy and stone extraction. Twenty-four hours post-ERCP the patient developed a fever, chills, bilirubinemia and elevated liver function tests. Ascending cholangitis was empirically treated using Ceftriaxone and Metronidazole. However, the patient remained febrile, with a diffusely tender abdomen and elevated inflammatory markers. A CT revealed a very small hypodense lesion in the seventh liver segment. Extended-spectrum beta-lactamase positive Klebsiella Pneumonia and Enterococcus Hirae were identified, and the antibiotics were switched to Imipenem and Cilastatin. The hypodense lesion in the liver increased to 1.85 cm and a new hypodense lesion was seen in the right psoas. At day 10 post-ERCP, the patient started having low back pain and difficulty walking. MRI revealed L4-L5 discitis with a large epidural abscess, spanning L1-S1 and compressing the spinal cord. Decompressive laminectomy of L5 was done and Klebsiella pneumonia was identified. Due to continued drainage from the wound, high fever, we performed a total body CT which revealed increased liver and iliopsoas abscess. Decompressive laminectomy was expanded to include L2-L4 and multiple irrigations were done. Gentamycin and Vancomycin containing polymethylmethacrylate beads were implanted locally and drainage catheters were placed before wound closure. Multidisciplinary panel discussion was performed, and it was decided to continue with a non invasive approach .

**Conclusions:**

Early recognition of complications and individualized therapy by a multi-disciplined team is important for managing post-ERCP septic complications. Particular attention should be given to adequate coverage by empiric antibiotics.

## Background

Endoscopic retrograde cholangiopancreatography (ERCP) is an increasingly applied endoscopic technique. It is used for both therapeutic (eg, bile duct stone removal) and diagnostic interventions in the bile and pancreatic ducts [1]. The latter include: tissue sampling from biliary or pancreatic lesions, sphincter of Oddi manometry, and diagnostic pancreatoscopy or cholangiography [2]. However, as less-invasive diagnostic tools, such as magnetic resonance cholangiopancreatography and endoscopic ultrasound have become widespread, the proportion of ERCP for therapeutic purposes increased from 66% in the early 1990s [3] to 89% in the late 2000s [4]. Therefore, ERCP has become largely performed for therapeutic interventions [5, 6].

Nonetheless, when compared to other endoscopic techniques, ERCP presents a higher potential for serious complications [7]. The major complications of ERCP include pancreatitis, cholangitis and duodenal perforation; and may be classified by site, timing and severity [8, 9]. Various rare complications have been reported as well, including gallstone ileus, liver abscess and splenic, hepatic and vascular trauma. Despite technological progress and scientific guidelines, ERCP-related morbidity and mortality have not decreased over time [4, 10, 11]. Many independent risk factors for post-ERCP complications have been identified including operator, hospital, method (eg sphincterotomy) and patient-related factors [1, 11].

A particularly morbid group of post-ERCP complications are infections [7], which are frequently caused by enteric bacteria [12, 13]. The most common septic complication is ascending cholangitis [14], which typically presents within 24 to 72 h post-ERCP [15, 16]. The cause is most often incomplete drainage of an infected and obstructed biliary system, which leads to elevated biliary pressure, and subsequently to biliary-venous reflux [17]. Clinical presentation may include the triad of Charcot or the Reynolds pentad, or their components, and may be associated with a liver abscess [18, 19]. The treatment for ascending cholangitis is based on decompression, antibiotics and supportive care.

Pyogenic liver abscess (PLA) is a life threatening complication of biliary tree infection post ERCP procedure,with mortality rate reach 12% [20], however Metastasis infection from a pyogenic liver abscess by hematogenous spread to other organs or spaces include epidural abscess or iliopsoas abscess is a potential complication. in our case we will present a rare case of spinal epidural abscess (SEA) originating from a PLA post-ERCP cholangitis.

## Case presentation

A 69-year-old man presented to the emergency department with right upper quadrant abdominal pain that started after a fatty meal. His medical history included diabetes mellitus, hypertension and ischemic heart disease. His surgical history included coronary artery bypass grafting, appendectomy and an inguinal hernia repair. He was hemodynamically stable and afebrile with obstructive jaundice.

Physical examination demonstrate a right upper quadrant tenderness, murphy sign was negative. Lab tests exhibited, normal leukocystes, increased hepatocellular enzymes (total bilirubin 2.6 mg/dl, direct bilirubin 1.17 mg/dl) and liver function test abnormalities (GGT 1255 U/L, ALT 480 U/L, AST 297 U/L, ALP 194 U/L). An abdominal Ultrasonography revealed fatty liver and a normal size gallbladder without thickened walls,with gallstones without bile duct dilation . Even though US imaging did not demonstrate signs of an inflamed gallbladder, the patient was considered to have cholecystitis with obstructive jaundice due to a convincing clinical presentation and remarkable right upper quadrant tenderness, he was admitted to our surgical department and managed conservatively with intravenous antibiotics and supportive care. The patient improved clinically,lab tests was improved also, bilirubinemia was decreased to 1.9/0.7 mg/dl and he was discharged; interval cholecystectomy was recommended following an endoscopic ultrasound (EUS).

**Fig. 1:**
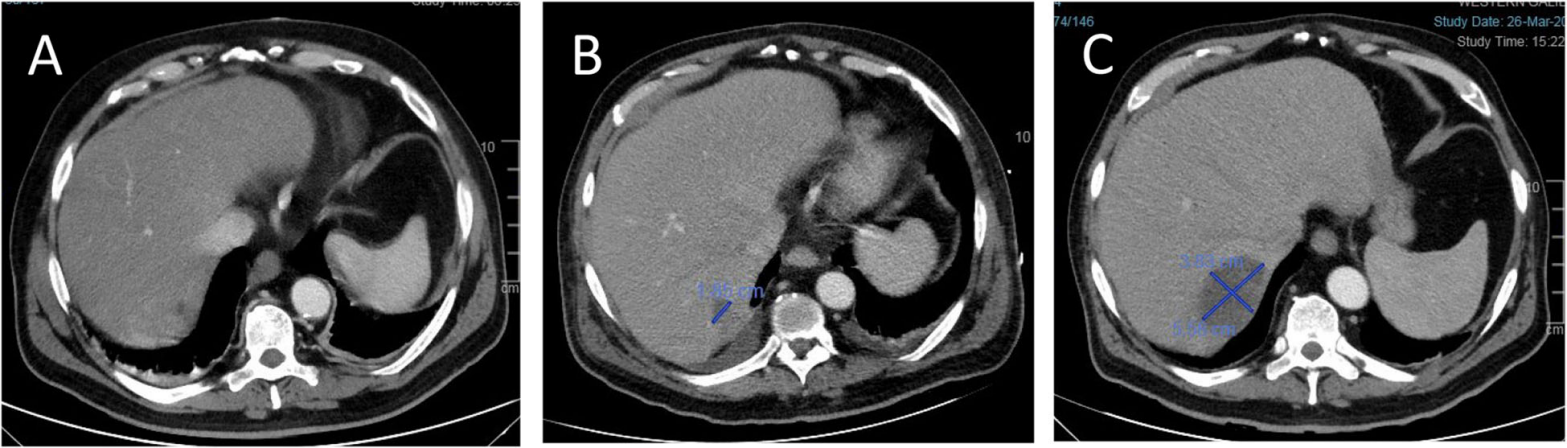
Liver abscess development on consecutive double contrast abdominal computed tomography scans. Scans were done on days 3 (**a**) 7 (**b**) and 26 (**c**) post-endoscopic retrograde cholangiopancreatography

Two weeks after discharge the patient underwent an EUS, which demonstrated a gallbladder with gallstones and sludge. Biliary duct dilation of up to 6.4 mm with 2 filling defects were also visualized. An ERCP was recommended and performed a week and a half later. A papillotomy and stone fragment extraction using a balloon catheter and a basket were performed. After the ERCP, the patient was admitted to our department for observation. Twenty-four hours post-ERCP the patient developed a fever (38 °C), chills, hyperbilirubinemia (2.66/1.16 mg/dl) and elevated liver function tests (AST 224 U/L, ALT 121 U/L, ALP 160 U/L, GGT 570 U/L). Ascending cholangitis was empirically treated using Ceftriaxone and Metronidazole. However, he continued to be febrile, confused and with chills. His physical examination was remarkable, his abdomen was diffusely tender and his lab tests reveal an increasing in inflammatory markers: C-reactive protein (CRP) 256 mg/l, leukocytosis 14(× 10 [3]/ul),with left shift 92% neutrophils. Also markedly hyperbilirubinemia was observed (total bilirubin 5.53 mg/dl), Blood cultures were drawn to rule out a viscous perforation. A computed tomography (CT) scan with double contrast of the abdomen and pelvis was done 3 days post-ERCP. A perforation was ruled out as no abdominal free air or fluids were found. Yet a very small hypodense lesion was found in the seventh liver segment (Fig. 1a).

**Fig. 2:**
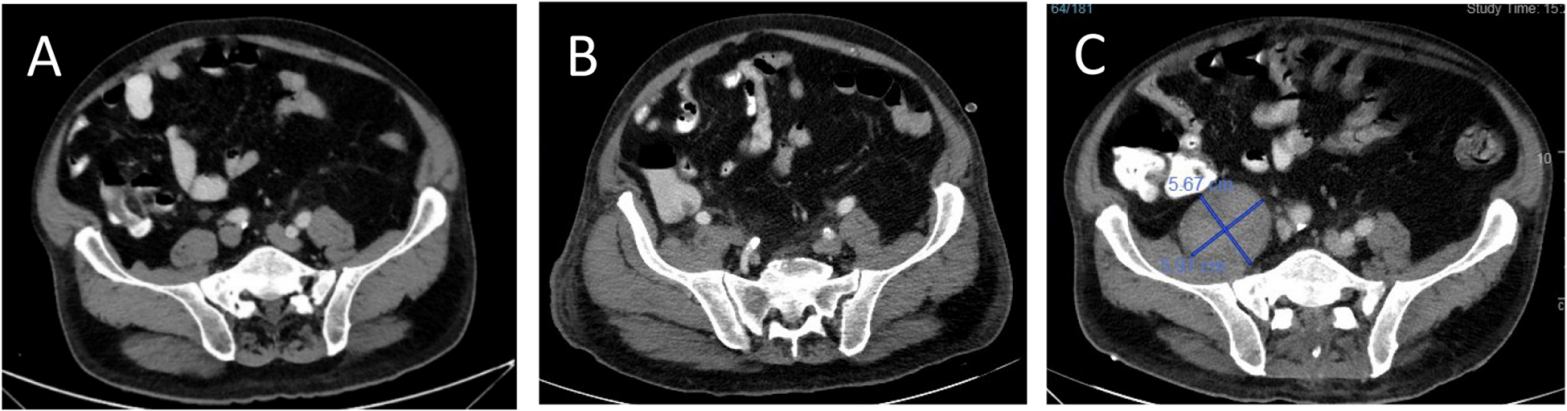
Psoas abscess development on consecutive double contrast abdominal computed tomography scans. Scans were done on days 3 (**a**) 7 (**b**) and 26 (**c**) post- endoscopic retrograde cholangiopancreatography ERCP

**Fig. 3:**
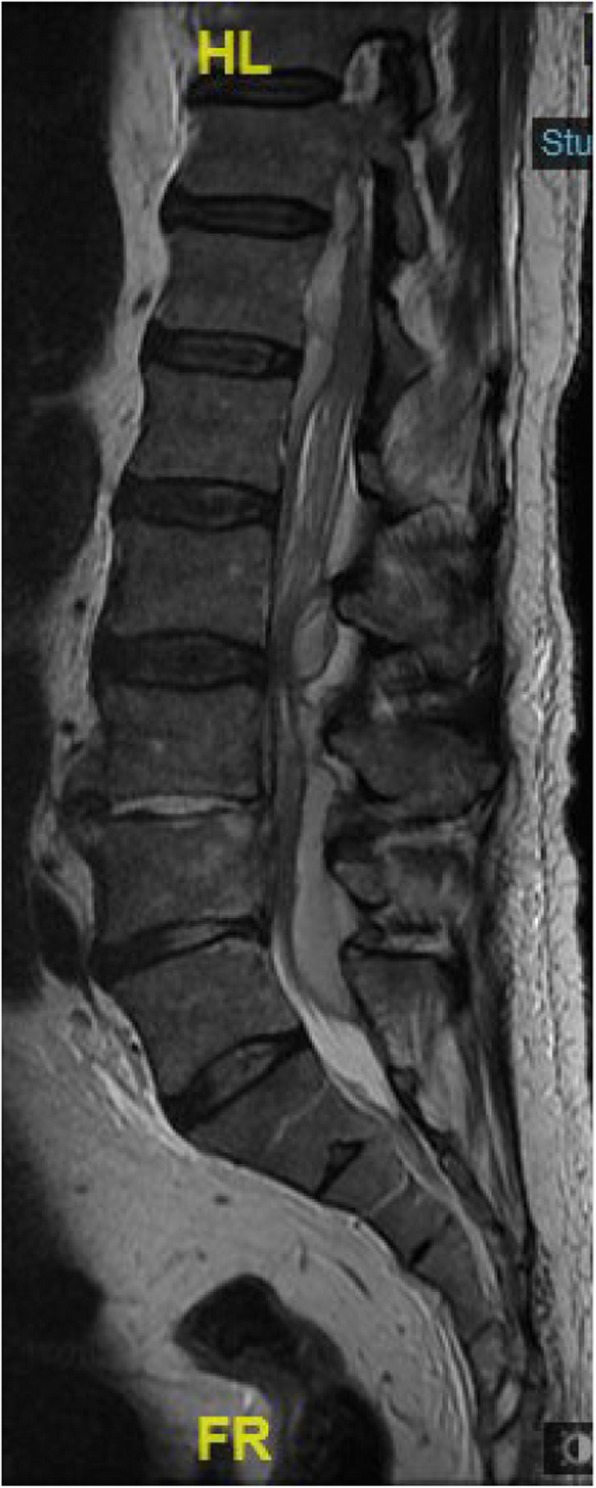
Spinal epidural abscess as seen on magnetic resonance imaging of the lumbar spine on day 19 post-endoscopic retrograde cholangiopancreatography

On day 6 post-ERCP, the antibiotic regimen was changed to Ertapenem and Vancomycin, to treat possible multi-drug resistant pathogens. Two days later, extended-spectrum beta-lactamase positive Klebsiella Pneumonia and Enterococcus Hirae were identified on blood cultures so antibiotics were switched to Imipenem and Cilastatin. Nonetheless, the patient continued to have abdominal pain. Upon examination, his abdomen was soft and distended, with no signs of peritonitis. CRP was elevated (160 mg/L), with mild leukocytosis (13 x 10e^3^/uL) and neutrophilia (83%). CT scan, with a double contrast of the abdomen and pelvis was repeated on day 7 post-ERCP revealing a hypodense irregular lesion that was found to increase in diameter, this finding was consistent with pyogenic liver abscess (Fig. 1b). Also, a hypodense lesion in the right psoas appeared (Fig. 2b), which was not seen on the first CT scan (Fig. 2a), it was decided to continue with antibiotic treatment without drainage. Two days after the antibiotic regimen was changed to Imipenem and Cilastatin, the abdominal pain subsided. He was afebrile and his liver function tests and bilirubin normalized. However, on day 10 post-ERCP, the patient began to complain about low back pain and difficulty walking. On physical examination, he had localized tenderness over the spinous processes of L4-L5, and reduced quadriceps femoris strength (3/5). A thoracolumbar spine magnetic resonance imaging (MRI) scan was recommended by a consulting orthopedic surgeon to evaluate for possible myelopathy or radiculopathy. MRI was performed on day 19 post ERCP, which revealed L4-L5 discitis,a large iliopsoas abscess on the right side,and other small abscess on the left side, an epidural abscess in front of spinal canal at level L1-D11 other one at level L2- S1 appearing to compress the spinal cord (Fig. 3). A diagnosis of spinal epidural abscess (SEA) with discitis and osteomyelitis was established. The patient was transferred to the orthopedic department and was urgently operated. Decompressive laminectomy of L5 was done and a large epidural abscess was drained and irrigated. Drainage catheters were placed, and the wound was closed in layers. Cultures from the pus were positive for Klebsiella pneumonia. Thus, IV antibiotics with polymyxin E and Gentamicin were initiated Post-operatively, and the patient’s back pain significantly subsided. Nonetheless, due to continued of serosanguineous drainage from the surgical incision, fever of 38.7^C^, CT total body was performed on day 7 postoperative to evaluate epidural and liver abscess, exhibited a 4.8 cm hypodense irregular lesion at segment 7 of the liver,epidural and psoas abscess were increased in size (Figs. 1c,2c). patient was taken back to the operating room for other evaluation. The decompressive laminectomy was expanded to include L2-L4, and multiple irrigations were done. Gentamycin and Vancomycin containing polymethylmethacrylate beads were implanted locally and drainage catheters were placed before wound closure. The patient was continued intravenous antibiotics with polymyxin E and Gentamicin. Two days later the patient was presented to the multidisciplinary panel (surgery and radiology) for further evaluation and discussion of liver and iliopsoas abscess treatment options. In light of the clinician and laboratory improvement it was decided to continue with a non invasive approach and to complete 6 weeks of antibiotic treatment. During the subsequent days the patient’s condition improved, the surgical incision was inspected without signs of infection or leak, and his lap tests normalized. Two months later,abdominal and spinal cord CT scan was performed as part of the follow up examination which assured almost complete regression of liver and iliopsoas abscess. An interval cholecystectomy was performed 6 weeks after discharging.

## Discussion and conclusions

In this case report, we presented a patient with multiple rare septic complications after a therapeutic ERCP. To our knowledge, this is the first time that this specific constellation of complications has been reported in the context of ERCP. Our patient presented with a PLA post ERCP procedure, predisposing factors for this infection are diabetes mellitus and post ERCP cholangitis [21]. empiric therapy was promptly started, bacteremia probably resulted in the subsequent septic complications, including a liver abscess. There is high rates of bacteremia in PLA which can lead to distant infection metastasis to other organs or spaces, especially in K.pneumonia pathogen [22]. Spinal epidural abscess (SEA) as metastatic infection originate from PLA still a rare complication, some case reports mentioned the association of a metastatic K.pneumoniae bacteremia and PLA to other spaces like cervical abscess or SEA [23]. SEAs have 5% mortality rate due to massive sepsis [24], and unfortunately the prognosis of SEA still poor due to delay in diagnosis, in our case early diagnosis of SEA was established and efficient treatment was initiated. Usually PLAs are polymicrobial with enteric facultative and anaerobic. The most commonly gram negative bacilli identified in PLAs are E.coli and Klebsiella pneumonia [25].

Another possible reason for the development of bacteremia in our patient was inadequate coverage by empiric antibiotics. In a study conducted in two German tertiary centers, empiric antibiotics did not cover the full biliary pathogen spectrum in 78% of the cases [26]. Furthermore, multi-drug resistant bacteria were isolated in 29% of the patients, including extended-spectrum beta-lactamase producing organisms. These findings raise the question as to whether empiric treatment for cholangitis, and specifically hospital-acquired cholangitis, should be modified to cover more pathogens or to routinely cover multi-drug resistant organisms. Presumably, earlier treatment of our patient with a better suited antibiotic regiment could have shortened or even prevented bacteremia and thereby reduced the risk for further septic complications.

In conclusion, we presented a patient with PLA caused by post ERCP cholangitis,subsequent with K.pneumoniae bacteremia and metastatic spread to SEA and iliopsoas [22]. He was treated with both medical and surgical treatment. A multidisciplinary treatment team of general surgeons, an infectious disease specialist, orthopedic surgeons and radiologists were needed to care for this patient. As ERCP is expected to become more common, it is important that all clinicians should be aware of the possibility of rare complications of this procedure therefore Early diagnosis of complications and prompt therapy should be key in the management of these patients.

## Data Availability

Data sharing is not applicable to this article as no datasets were generated or analysed during the current study.

## Abbreviations

ERCP: Endoscopic retrograde cholangiopancreatography
CRP: C-reactive protein
CT: Computed tomography
MRI: Magnetic resonance imaging
